# Navigating adulthood with maple syrup urine disease: patient and caregiver perspectives on healthcare transition and independent living

**DOI:** 10.1186/s13023-026-04383-6

**Published:** 2026-07-25

**Authors:** Jessica I. Gold, Megan Grabill, Karen Dolins, Melissa Basile, Bat-Zion Hose, Rebecca Ganetzky

**Affiliations:** 1 Division of Medical Genetics, Department of Pediatrics, Cohen Children’s Medical Center, Northwell, New Hyde Park, NY USA; 2 Institute of Health System Science, Feinstein Institutes for Medical Research, Northwell, Manhasset, NY USA; 3 Department of Family Medicine and Community Health, Perelman School of Medicine, University of Pennsylvania, Philadelphia, PA USA; 4 Department of Anthropology, University of California Los Angeles, Los Angeles, CA USA; 5 Teachers College, Columbia University, New York, NY USA; 6 Research Lead, MSUD Family Support Group, New York, NY USA; 7 MedStar Health National Center for Human Factors in Healthcare, Washington, DC USA; 8 Georgetown University School of Medicine, Washington, DC USA; 9 Perelman School of Medicine, Division of Human Genetics, University of Philadelphia, Children’s Hospital of Philadelphia, Philadelphia, PA USA; 10 Division of Medical Genetics | Northwell Health, 225 Community Drive, Great Neck, NY 11021 USA

**Keywords:** Adult metabolic medicine, Health care transition, Maple syrup urine disease, Long-term outcomes, Qualitative research

## Abstract

**Background:**

Medical advances in diagnosis and management have increased the life expectancy of many pediatric-onset rare diseases, including inherited metabolic disorders (IMDs). Historically fatal childhood conditions are now manageable chronic illnesses, with over 90% of individuals with IMD reaching adulthood. As more individuals with IMDs reach adulthood (age 18 years), there is a new imperative for health care transition planning that promotes developmentally-appropriate skills for medical self-management and independent living. Knowledge gaps on the long-term disease-related outcomes limit our ability to provide evidence-based resources for transition. Maple Syrup Urine Disease (MSUD) is an IMD with a global incidence of 1:185,000. It is increasingly diagnosed through population-level newborn screening, which allows for early treatment with dietary restrictions of branched chain amino acids and urgent intervention during episodes of catabolic stress. Due to the success of newborn screening programs, there is a growing population of adults with MSUD, making it a model for exploring the current state of transition and adult outcomes in IMDs.

**Results:**

We used a qualitative case-study approach to assess the lived experiences of adults with MSUD (*n* = 10) or caregivers to adults with MSUD (*n* = 8). Analysis of their semi-structured interviews identified common themes including: (1) poor, non-standardized health care transition; (2) less emphasis on dietary management; (3) changing symptomatology with new presentations of fatigue, brain fog, and panic attacks; (3) emerging concerns related to adult health care, including comorbid adult-onset conditions, mental health, and lack of knowledgeable adult providers; (4) wide-ranging functional outcomes for education, employment, and financial independence; and (5) attainment of new milestones including romantic partnership and parenthood; and (6) greater concern about the supports needed to achieve independent living.

**Conclusions:**

Individuals with MSUD are reaching milestones of adulthood and have new priorities for their healthcare and lifestyle. Evidence-based healthcare transition is needed to support their transition to independent living.

## Background

Modern medical advances have transformed many childhood-onset disorders to manageable chronic conditions with extended life expectancy and improved clinical and patient-reported outcomes [1–4]. Thus, more adolescents and young adults (AYA, individuals aged 14–26 years) with special healthcare needs are reaching the critical transition period to adulthood with corresponding risks of lapses of healthcare and psychosocial supports. Transition-related challenges are magnified for AYA with rare disease due to the complexity of these disorders, frequent comorbid intellectual disability, lack of trained adult providers, and limited coordination between pediatric and adult care systems [5, 6]. The current healthcare transition (HCT), *the planned*,* purposeful movement from pediatric to adult-centered healthcare*, for this population is non-standardized and occurs infrequently in the United States [6, 7]. Transition should be developmentally-appropriate and address medical self-management, independent living skills, educational or vocational rehabilitation, financial assistance, and decision-making support). The transition process usually begins at age 14 years with a graduated, individualized approach to independence [8]. Historically, pediatricians have been responsible for transition. With increased life expectancies for many complex childhood-onset conditions, subspeciality providers are now initiating more transition planning [4]. However, our ability to design and implement effective HCT is limited by knowledge gaps on social and medical outcomes for adults with childhood-onset rare disease.

Inherited metabolic disorders (IMD) are a subset of rare disease with a rapidly growing young adult population due to the expansion of newborn screening programs in the early 2000s [9]. Though individually rare, the collective incidence of all IMD range from 1:800 to 1:2500 [10]. Population-based screening programs have led to early diagnosis and treatment, transforming historically fatal childhood diseases to manageable chronic conditions [11, 12]. Recent estimates suggest that over 90% of individuals with IMD are now reaching adulthood [1, 13]. However, many face barriers in achieving social and medical autonomy due to the complexity of care for their multisystemic conditions and high prevalence of intellectual or learning disabilities. IMD require multidisciplinary management that may include strict dietary restrictions or specialty medications, and affected individuals must balance their medical needs with the general demands of daily living [12, 14, 15]. Additionally, IMD are among the first rare diseases for which therapeutics have been leveraged to change the natural course of these disorders, suggesting a road map for other disorders in the era of emerging gene therapy [16–18]. As pediatric-onset IMD were historically confined to childhood, the lived experience of adults with IMD, including their transition from adolescence to adulthood, is largely unknown. Exploring the current status of adults with IMD, including their experiences with education, vocation, romantic partnerships, and navigating the transition to adult-oriented healthcare, offers insights to guide HCT for AYA with chronic childhood-onset conditions.

Among the IMD, Maple Syrup Urine Disease (MSUD) provides a representative population for how adults with pediatric-onset rare disease navigate the transition to adulthood. This autosomal recessive disorder is caused by decreased activity of the branched chain α-ketoacid dehydrogenase, the enzyme responsible for catabolism of isoleucine, leucine, and valine [19]. Leucine and its conjugate α-ketoacid are specifically neurotoxic, and its accumulation can lead to acute symptoms of cerebral edema, encephalopathy and death, as well as cause chronic neurodevelopmental symptoms such as poor feeding, intellectual impairment, and neuropsychiatric differences [20]. Treatment is lifelong and involves strictly limiting dietary intake of branched chain amino acids, emergently managing catabolic stressors, including illness, pregnancy, and prolonged fasting, and, for some individuals, liver transplantation to partially restore branched chain amino acid metabolism. As the worst sequalae are preventable through early detection and treatment, population screening of newborns for MSUD has been recommended since 2005 [21]. For many affected individuals, neurocognitive differences persist despite early intervention, potentially impacting their ability to obtain milestones of independent adulthood [20, 22]. HCT may alleviate these challenges, yet it is offered to few adolescents with MSUD [23]. 

This study used qualitative methods to describe the experiences of being an adult with MSUD. It expanded on challenges previously identified for adolescents with MSUD – dietary adherence, maintaining relationships, and frustration with continued medical care in pediatric spaces [23], by incorporating more milestones of adulthood including romantic partnership, reproductive planning, educational and vocational attainment, and financial independence. Experience with HCT and obtaining medical self-management skills was investigated in detail, as our participants should have completed this process. Emerging themes demonstrated (1) an increasingly mature perspective on managing chronic disease with less emphasis on the burdens of diet, (2) greater concern about gaps in HCT readiness, including limited supports needed to achieve independent living, and (3) wide ranging functional outcomes for education, employment, romantic partnership, parenthood, and financial independence. This research recognizes new priorities for HCT that centers social needs alongside medical self-management skills.

## Materials

### Setting and participants

Participants for this study were initially recruited for a larger, international study on neurocognitive outcomes in adults with MSUD [14]. Recruitment occurred through the newsletter of the MSUD Family Support Group (FSG), social media, and informational meetings with the young adult members of the MSUD FSG. During the initial consent process, subjects indicated their interest in participating in 1–2 hour-long semi-structured interviews. Subjects also volunteered contact information for a childhood caregiver who would be willing to partake in the qualitative aim. A purposive sampling method was used to achieve a diverse sample of adults and caregivers regarding race/ethnicity, age, geographic location, and neurocognitive ability (Table 1) [24]. Affected adults were required to be older than 21 years for greater exposure to the transition process. Interviews were conducted in English, so non-English speakers were excluded from enrollment. Ten affected adults aged 23–39 years-old and 8 caregivers to adults aged 21–40 years-old participated in the study. Our purposive sample strategy prioritized enrolling caregivers to adults with intellectual disability. Thus, only two caregiver-adult dyads were included. Two caregivers had multiple adult children with MSUD. Table 1 summarizes the demographics for the affected adults who were represented by self-report or caregiver report. The sample size was dependent on data saturation and was anticipated to be 15–25 based on literature review [23, 25, 26]. Participants were contacted by telephone or email. Verbal informed consent was obtained from all participants. This study was approved by the Children’s Hospital of Philadelphia IRB (IRB#21-18443).

**Table 1:** Demographics of affected participants

Gender, *n* (%)	
Male	6 (31.5)
Female	13 (68.4)
Age, mean (SD)	32.8 (6)
Race/Ethnicity, n (%)	
Asian	1 (5.3)
Black	1 (5.3)
White/Hispanic	2 (10.6)
White/non-Hispanic	15 (78.9)
Geographic Location	
Canada	3 (15.8)
Europe	2 (10.6)
United States	14 (73.7)
Residence, n (%)	
Alone	2 (10.5)
Domestic Partnership	7 (36.8)
Parents	9 (47.3)
Marital Status, n(%)	
Single	11 (57.9)
Married	7 (36.8)
Divorced	1 (5.3)
Children, n (%)	4 (21.1)
Education	
Some High School	1 (5.3)
High School Diploma	3 (15.8)
Some College	4 (21.1)
College Degree	5 (26.3)
Graduate Degree	6 (31.6)
Employment, n (%)	
Full-time employment	8 (42.1)
Part-time employment	8 (42.1)
Unemployed	1 (5.3)
Student	2 (10.5)
Liver Transplant	3 (15.8)
Symptomatic at Diagnosis	16 (84.2)

### Data collection

Two semi-structured interview guides were developed for either affected adults or caregivers. These guides were informed by previously performed qualitative studies with adolescents with MSUD and a broad review of IMD and HCT literature [23]. Interview guide revisions occurred through discussion among experts in qualitative research methods, clinical care of MSUD, and HCT. The MSUD Adult Interview consisted of 32 open-ended questions and was developed to explore the experience of transitioning from adolescence to adulthood with MSUD, while the Caregiver Interview consisted of 36 open-ended questions addressing the caregiver perspective on HCT and attaining adult milestones. Questions were divided into 6 categories based on domains emphasized in HCT [8]: (1) Life experiences with MSUD; (2) Relationships and Reproductive health; (3) Education; (4) Employment; (5) Independent living and finances; and (6) Medical experiences and HCT. Telephone interviews were conducted by a single researcher from June to October 2022. All interviews occurred in private spaces and were audio-recorded with participants’ consent. Audio recordings were then sent to the University of Pennsylvania’s Mixed Methods Research Lab where they were transcribed by a HIPPA-compliant service and de-identified.

### Data analysis

The codebook was developed by two members of the research team using a modified grounded-theory approach [27, 28]. Transcript analysis using this codebook was supported by NVivo software (http://www.qsrinternational.com). The same two members of the research team completed three rounds of inter-rater reliability with κ = 0.8, using double coding on 17% of the transcripts (*n* = 3). Coding discrepancies was discussed and resolved in each session. The remaining transcripts were divided evenly between the two research team members and coded separately.

## Results

### Qualitative overview with central themes

The design of our interview guide fulfilled two main purposes. First, we wanted to explore how MSUD impacts adulthood. Participants reflected on their current living situation, employment, romantic relationships, and medical management. The second goal was to recount the process of making it to adulthood by describing necessary supports, graduated approaches to independence, and HCT-related planning. Together, our themes depict adult lifestyles, but also illustrate a path forward for the next generation of adolescents with MSUD. Table 2 includes themes with illustrative quotes. Overall, our interviews demonstrated a wide spectrum of functioning for adults with MSUD from complete independence to continued reliance on caregivers for medical, financial, and living assistance.

**Table 2:** Supportive illustrative quotations: adults with MSUD and their caregivers

Interview Category Theme	
Medical Experiences and HCT	
*Limited formal transition planning*	“I feel like maybe it almost delegitimizes MSUD because it makes it seem like it’s something that affects you mostly when you’re younger because you’re seeing a doctor that primarily deals with children.”
*Mental Health Burdens*	“Is this the MSUD or is this depression? You never know. I mean, the bottom line is you don’t know. There’s no way to tease that out. But there’s an awareness of the need to separate that out.” “I started having really bad depression, where I just didn’t want to drink my formula, I didn’t want to eat, I didn’t want to do anything. And I started becoming suicidal and everything. I slowly was making myself sick by not drinking my formula. It was the first year where I didn’t really pay attention or care enough to drink my formula because I was away from home. I would come home and basically be hospitalized for a few days till a week, on and off that year because I would basically hurt myself by not drinking the formula.”
Life Experiences with MSUD	
*Eventual acceptance of dietary restrictions*	“I’ve never struggled with diet. I think it’s maybe a little easier for me. I have no desire for anything. I’m on a purely vegan diet and I’ve always tried to really watch what I’m consuming.” “It’s just being consistent and knowing what you’re doing. That’s the sacrifice, but you got make it.”
Relationships and Reproductive Health	
*Relationships impart support*	“…part of his eat to live not live to eat mentality helped us get around whatever meals would look quite for us because it wasn’t like a focal point for him the way it is for some people. So, it did provide me a lot of flexibility and safety and security like I never felt like he was judging me. He would actually make my protein shake. I remember when we were first dating, I wasn’t feeling good and I needed to have my protein shake and he did the powders and went to the water fountain and he wasn’t weirded out, so it was a good sign.” “So, I said to him, "What do you think my [leucine] levels are? He said, "Well, they’re probably lower normal knowing you,” and last week he was going through the stress. I could tell his levels were off. So, we came to an agreement. When one of our levels are off, we go by the brain of the person whose head is on”
*Challenges in judging relationship quality*	“She had a serious relationship with someone that we didn’t know him. There were a lot of red flags to the relationship, so we were discouraging it, but more importantly her therapist was discouraging it. And so, she’s still trying to move beyond it. She desperately wants to feel loved and he told her he loved her. So that was very compelling” “It’s very challenging because they tell each other, they say, oh he cheated on you. They just talk, they over exaggerate, they’re like middle schoolers. It is kind of a middle school level.”
Education	
*Accommodations make advanced education achievable*	“If your kid is able to handle going away to school, let them do it, because that could be the only time that they live independently from you. Give them that opportunity if you can.”’ “I just wish that I was able to have the [ADHD] medication that everyone else does and that I wouldn’t exist in this weird… It’s kind of like a weird headspace having ADHD. And it’s almost like you’re watching yourself do these things and you can’t make it stop which probably sounds really weird, but it’s very challenging and I know that people with MSUD have mental health issues and ADHD and whatever else. And I feel like I’m at a detriment, because I also have these other coexisting conditions. So, it’s kind of like a perfect storm.” “I signed up for at disability services at my University. I didn’t actually ever really use it. My mom basically just said, “You want to have something in place in case something happens and you’re delirious and you need to reschedule an exam or miss it or something like that. You have some paperwork that says you’re not going to get a zero or something.”
Employment	
*Disclosure to employers is a difficult decision*	“ [Patient] did a lot of babysitting work last year and there was one job where she had to drive the kids around. She had a car accident with the kids in the car and thankfully everyone was fine, it was only the car that was damaged, but the mom did end up letting her go and I said to [Patient], "Did you explain to the mom that sometimes your levels are high and it’s not safe for you to drive?” She said, “No. If I did that, no one would hire me.” So that’s a huge challenge to have this disease that if you feel like you disclose, why would anyone hire you? And yet if you don’t disclose, look what happens? “Nobody really wants to give him a chance. So, I think that’s the most difficult part. Just don’t wanna hire anybody who has challenges” “Don’t let your MSUD be an impediment, but don’t let your health fall to the wayside either. I think that’s super important, because as we get older, we can think, oh, hey, I’ve made it to this stage, I’m doing well, but you never know what tomorrow will bring and if somehow your MSUD will start affecting you more. And if there are things that you can do to try to ensure that maybe you’ll hopefully be a little healthier, then do that even if it means that… I don’t really know how to put it, even if it means that you have a job that you have to work a few less hours or something, just remember that your health is paramount.”
Independent living skills	
*Wide spectrum of support needed for daily living*	“We want him to be an adult to have the freedom somewhat of an adult yet he does need monitoring. From an early age starting to teach kids everything that has to do with independence, doing their own laundry, the foods, being responsible for themselves. If anything, I feel like maybe we did too much for him that’s why he was never great at compliance. I always felt that. I don’t know what if he’d been more independent in terms of laundry and stuff like that had I done anything different.” “He lives on his own. Before his transplant, we felt he could not have done that. He did get his driver’s license before transplant, but I was always nervous because I felt that if he wasn’t feeling well or he didn’t have enough calories, his focus would change. I only allowed him to drive small distances but now with transplant I feel pretty confident.” “She’s very intelligent. She’s smart, she communicates well, she writes well. But she really lacks skills financially, because I think I mentioned in some of the previous surveys, she can’t really do her budget. She’s trying and she’s getting better. But she can just get in with these people friendship-wise that she trusts and tells them everything about her life in the beginning whether it’s financial, personal, [past] history of her health problem. More likely than not these people she meets tend to take advantage of her emotionally and financially. She pretty much drained her bank account quite a few times, but now we’re being more cautious as to how we supplement, we’ll pay directly rather than give her assets more than what she earns or through SSI.”

### Preparation for adulthood - healthcare transition

Despite an international call for metabolic clinicians to lead transition due to their greater disease knowledge, long-standing therapeutic relationships, and multidisciplinary approach [7], only one participant discussed transition planning with their metabolic provider. Households that successfully helped their children become responsible for MSUD care accomplished this by gradually introducing more responsibility, without formal HCT planning. As one caregiver explained: “*It’s just been a process over the years that we’ve just -- it’s just like happened naturally. But I don’t remember ever sitting down with a healthcare professional and talking about it.”* It was not uncommon for caregivers to continue to manage insurance and finances well into adulthood.

Without a formal transition process, there was a wide range of independent medical management among adults with MSUD. Eight out of 10 adults relied on their caregivers to schedule appointments, pick up formula, and measure dosages, while seven split the responsibilities, often either attending appointments by themselves or taking care of their own formula. Three adults reported complete independence in medical management. The typical first medical responsibility was preparing their own formula, which started around ages 8–10 years. Dietary self-management tended to be the most common topic of transition planning with few resources provided for the other aspects of independent adulthood. For liver transplant recipients, compliance with immunosuppression was the primary transition topic.

One challenge in transition planning was the lack of clinicians trained to care for adults with MSUD. All participants reported close relationships with their metabolic teams, who have provided lifelong care. However, all ten adults with MSUD had reservations about their continued care in pediatric spaces. One shared: “*I feel like maybe it almost delegitimizes MSUD because it makes it seem like it’s something that affects you mostly when you’re younger because you’re seeing a doctor that primarily deals with children. […] Sometimes I feel like as an adult with MSUD because these doctors are so used to dealing with children that they tend to speak to us like we are children and we’re full-grown adults… We deserve to be treated with respect and dignity*,* not talked down to.”* Adults expressed concerns about the lack of knowledge on aging with MSUD and about their metabolism providers’ ability to balance MSUD management with chronic adult-onset conditions. One participant expressed that “*In my opinion*,* it matters very little whether I’m at [Hospital 1] or [Children’s Hospital]*,* in that department. All my concerns are as novel probably at [Hospital 1] as they are at [Children’s Hospital].”* Both caregivers and adults felt that MSUD was used to explain any health issue, without adequate exploration into other causes.

Seven out of 10 adults with MSUD noted changes in their MSUD symptoms as they aged. Starting in their late teens, metabolic decompensations presented as headaches, fatigue, panic attacks, and brain fog, rather than their earlier presentations with predominant somatic symptoms, dysarthria, and ataxia. *“I don’t really lose balance or anything like that. Now it’s more like*,* I just feel out of it. You just feel like a fog*,* you don’t feel yourself.”* Three adults described decompensations that were triggered by emotional stress, such as high-pressure situations at work rather than a physical stressor like illness or fasting. One participant was concerned that her metabolic clinicians were not adequately assessing presenting symptoms for adults. She suggested that clinicians could talk to patients, *“more frequently when they have an episode to figure out what their symptoms are as they’re getting older because their symptoms just change…. The more they hone down on what their adult symptoms are*,* the easier it will be for the adult to figure out when they’re sick.”* These knowledge gaps of the adult MSUD spectrum could lead to inadequate transition planning.

### Dietary management

The strain of lifelong compliance to strict dietary restriction has been previously reported for adolescents with MSUD [23]. In contrast, our adults reported both personal and societal acceptance for their diet: “*There’s not as much of a social stigma around restrictive diets these days because a lot of people are gluten-free or lactose intolerant or vegan.”* Several adults described a moment of personal acceptance of their low-protein diet. These revelations tended to occur in their late teens to early twenties. Seven adults began to notice the trade-offs between dietary noncompliance and challenges in education, vocation, or intrapersonal relationships. One said: *“I was mad at myself because [my GPA] ended up at 3.1 and I wasn’t where I thought I would be by the end of freshman year. But when I got home and I found out what my levels had been*,* it dawned on me that I was just making not great choices at the dining hall. I got my levels down to 3 [mg/dL] and then the rest of college was great.”* Despite personal acceptance, three adults voiced concern about how the restrictive diet for MSUD impacts their overall health: *“When I see the dietician*,* they’re not thinking about necessarily diabetes or heart health or whatever with MSUD. They’re thinking about*,* you have MSUD. And so*,* you need this diet*,* and then I’m overweight and I need to not have all the sugar and fat. And then you look at all the sodium in all the low protein products and I’m like*,* this is great. I already stressed enough about what’s in food and there’s all these mixed messages and there’s nobody here who’s like*,* how do we look at all these things and put together a plan that you could actually live with.”*

### Mental health

The impact of mood on medical compliance was a new theme from our interviews. Four caregivers and two adults reported that depression led to significant differences in their diet or ability to self-manage their MSUD. Both adults shared that they stopped following their protein-restricted diet as a method of self-harm. One shared: *“I started having really bad depression*,* where I just didn’t want to drink my formula*,* I didn’t want to eat*,* I didn’t want to do anything. And I started becoming suicidal and everything. I slowly was making myself sick by not drinking my formula.”* Five out of 10 adults had received intensive or inpatient care for psychiatric illness. Most other participants noted a history of depression, anxiety, panic attacks or post-traumatic stress disorder. Difficulty finding a mental health professional was a common barrier to obtaining care.

### Impact on relationships

Social: Five out of ten adults described their friends as sources of support and potential advocates, with one advising: “*Make sure that your friends know what to do in case you ever exhibit any kind of issues*.” For those whose MSUD was a hidden disability, discretion about their diagnosis was possible and often preferred. Adults with more visible disabilities reported more adversity, including the impact of prolonged school absences and mental health concerns on developing social skills. Four caregivers expressed worry over their child’s judgement in choosing friends and shared examples of exploitative friendships: “*More likely than not these people she meets tend to take advantage of her emotionally and financially. She pretty much drained her bank account quite a few times*.” Childhood caregiver accommodations, such as chaperoning class trips to ensure dietary adherence, did not readily translate to adulthood, leaving caregivers only able to offer advice.

Romantic: Romantic relationships in our sample population ran the gambit from single to married to divorced. Both participants and their caregivers expressed their desire for romantic partnership as a symbol of normalcy. Those in relationships tended to disclose their MSUD diagnosis prior to or during a first date, either to demonstrate trust or to screen for incompatibility: “*I don’t want to say that [disclosure] was a test, but if I’m telling you this and you can’t accept it then you’re not right for me anyway*.” One participant noted her future spouse’s willingness to mix her formula as a significant moment early in their relationship. Five caregivers to adults with more significant disabilities, particularly those affecting decision-making and independence, expressed more apprehension around dating. Multiple caregivers recounted instances where their children have engaged in romantic relationships that were concerning for inappropriate or predatory behavior. Caregivers often intervened to help set boundaries, especially when technology is involved: *“We’ve had to talk about pictures. I know she was talking to this boyfriend through the screen when she would be in a state of undress. I think she intellectually knows that this is dangerous*,* that people can capture these images and they can be out there forever*,* but that doesn’t translate into her behavior.”* However, they also recognized the limits of their influence on their adult children.

Reproductive Health: Conversations about reproduction frequently referenced genetic testing. Some adults did not plan to pursue genetic testing prior to pregnancy as they were comfortable managing MSUD in potential offspring. One adult’s spouse opted for genetic screening due to concerns over work-life balance: *“I don’t think I can be a mom with kids with your disease. I think it would be just too hard because you and I both have careers.”* Siblings of affected adults also underwent genetic screening – which was reported to be influenced by the burden of MSUD on the entire family. MSUD also affected some participants’ interest in having children. Four adults noted their continued reliance on their parents and expressed concerns about their ability to parent: *“I feel like taking care of a child would drain me to no end.”* One adult felt that managing a pregnancy with MSUD would be too dangerous. Other participants spoke about their desire to be pregnant. Two adults relayed challenges with infertility and spontaneous abortions, which caused a negative impact on their mood.

Three participants had successful pregnancies and spoke about their experience. Pregnancy required meticulous management, primarily by dietitians, but was overall safe. One adult experienced post-partum hyperleucinosis that required hospital admission. Another patient’s pregnancy was complicated by gestational diabetes that required multidisciplinary collaboration between metabolism, endocrinology, and maternal fetal medicine. Metabolism led the coordination due the unique characteristics of MSUD, include the risk of post-partum complications. One participant’s pregnancy was complicated by a congenital defect in her fetus. She shared feelings of guilt and frustration over knowledge gaps on MSUD’s potential impact on embryogenesis.

### Academic competencies

Participant experience with academics varied widely. Educational attainment by participants with MSUD ranged from partial high school education to completion of a PhD. Affected adults shared academic experiences from elementary school through college. Challenges at school arose from both learning differences and MSUD-related medical complications. Participants noted the difficulty of maintaining academic performance while dealing with prolonged admissions for acute metabolic crises. Four adults and two caregivers recognized the impact of co-occurring ADHD, further impeding academic success. One participant noted *“feeling like I’m at a detriment*,* because I also have these other coexisting issues [MSUD*,* ADHD*,* and mental health]. So*,* it’s kind of like a perfect storm*,* but I wish it wasn’t.”*

There was no consensus about disclosing MSUD to teachers. One caregiver reported increased support from teachers after explaining MSUD. However, one participant noted the risk of increased social stigma: *“In high school*,* we were learning about genetics in biology. And the teacher brought up MSUD and how they said you could smell of maple syrup… he used the word retarded. So*,* after the class*,* I walked up to him and said*,* “I have this disorder. You pretty much told me I was retarded and if anyone else found out now they’re going to think I’m retarded.” And he was so embarrassed…. And he’s like*,* “Do you want to do a case study and explain what it is?” I’m like “No*,* you just told everyone all these bad things that kids are already pick on me. If I give a moment*,* it would be like giving more material to pick on me for.” So*,* I made sure no one ever found out.”*

Academic accommodations were common through primary schooling. Of the eight caregivers interviewed, seven mentioned that their child had an Individualized Education Plan (IEP) that covered MSUD and other medical needs, such as ADHD, autism, dyslexia, and cerebral palsy. Accommodations ranged from reinforcement of dietary requirements to extended time and modified testing to enrollment in a specialized school for children with disabilities. Participants did not report barriers in obtaining IEPs or adapting them when needed.

Our interview guide specifically asked about enrollment in post-high school transitional education or vocational programs. Six of eight caregivers mentioned that their child participated these programs, which ranged from a vocational track within the school system to a multi-year residential program, which allowed a young adult with MSUD to gain independent living skills in a monitored environment. Two caregivers reported that they received no guidance about transitional education: *“I don’t think the school does a very good job in helping kids who have any kind of disability. You graduate and then you are kinda on your own.”*

College Experience: Overall, thirteen affected adults attended college – eight from our adult interviews and five from our caregiver interviews. Of these, three lived independently on their college campus. One participant struggled with the transition to college and returned home after her first semester. For seven adults, increased independence in college complicated their dietary management, with challenges in adhering to their low-protein restrictions and ensuring that they ate regular meals. One reflected: “*In high school*,* my mom was still doing things for me*,* still calling me to eat. When I got to college it was upon myself to do all of that…and it was never easy to remember to eat.”* For one affected adult, parents eased the transition to college by making and delivering individual servings of formula. Affected adults tended to be more discrete about their MSUD in college, though one adult registered with Disability Services as a proactive measure against MSUD complications, explaining: *“My mom basically said “You want to have something in place in case something happens and you’re delirious or whatever and you need to reschedule an exam. You have some paperwork that says you’re not going to get a zero.”* One caregiver recognized the overlap between substance use and the symptoms of a metabolic decompensation: *“We met with the health services and explained everything. There was a banner that ran across the top of his record that said don’t assume he’s drunk or high if you see unsteady gait*,* dizziness… call his doctor or his parent.”*

### Employment

As with education, vocation varied widely among the adults with MSUD. Some were unemployed, some had part-time jobs in food service, others were pursuing careers as librarians, financial assistants, or scientists. Skills needed for employment ranged from manual labor to operating heavy machinery, such as forklifts, to extensive case management, including patient care. Accordingly, workplace disclosure and accommodations were highly dependent on the individual needs for each job. One participant adamantly denied any reason to disclose, saying: *“I feel it doesn’t really impact me at the end of the day.”* Some participants were able to successful disclose their diagnosis without consequence: *“They understand I have some medical issues and they know I have my limits too. So they’re very*,* very nice about it.”* Proactive communication about emergent medical management was critical. One caregiver described an incident where her child required a prolonged hospital admission that was not adequately communicated to her boss and led to termination. Concurrently, the caregiver recognizes that disclosure can be harmful: “*So that’s a huge challenge to have this disease if you feel like you disclose, why would anyone hire you? And yet, if you don’t disclose, look what happens?”*

### Independent living skills in adulthood

Driving: Fourteen adults with MSUD had a driver’s license with one adult currently studying for the test. Among adults, confidence behind the wheel varied greatly. One patient reported growing recognition of delayed reaction time and creating a personal rule equating driving impairment with seeking emergent medical care. Five caregivers had reservations about their child driving due to concerns about reactivity and brain fog. Access to transportation facilitated independence and employment, and adults without licenses frequently used rideshare services for work.

Living Situation: The caregiver interviews were split evenly between those who lived with their adult children and those whose children lived independently. Affected adult competencies with daily tasks such as chores, food preparation, and financial management varied greatly in these interviews. Four caregivers felt that their children would never be able to live fully independently, and had taken legal and financial measures to ensure that their children would continue to receive adequate care. This included filing for legal guardianship, power of attorney, and establishing a trust. Five of eight caregivers mentioned feeling apprehensive about their child’s options for care after the caregiver’s death.

Affected adults had varied living situations and levels of independence. Some lived independently or with spouses, while others lived with their parents or in apartments or suites connected to their parent’s house. Approaches to mealtime varied. For those living at home or semi-independently, it was not uncommon for caregivers to help with food preparation for the adult. Other times, households chose to cook separate dinners. However, some households attempted to provide low-protein options alongside more standard fare. One participant mentioned that there was occasional tension between her and her spouse, as he was not good at making meals that she could also eat: *“If my husband’s the one that decides to make dinner, he never makes something low protein for me unless I explicitly asked him, it’s usually me making myself something separate and if I’m too tired to make them dinner, usually I’m too tired to make myself dinner too kind of thing and it’s just something that I don’t like to have to explicitly state.”* Affected adults and caregivers both mentioned that fatigue made it challenging for adults with MSUD to complete daily tasks. One caregiver mentioned that all three of her children with MSUD struggled with their energy levels and had grown accustomed to napping. One affected adult with unaffected children reported that fatigue occasionally impacted her ability to parent, including delaying meals or homework supervision.

## Discussion

This study is among the first to capture the lived experience of being an adult with an IMD. Our interview guides focused on the topics that are typically addressed through transition planning as representations of milestones for adulthoods. Similar to our previous quantitative study [22], our interviews illustrated the wide range of functional outcomes experienced by adults with MSUD. Some adults with MSUD are successfully completing college, living independently, enjoying romantic partnerships, and finding full-time employment. There are also adults with MSUD who are unable to obtain these markers of independent adulthood, who continue living with caregivers and need assistance with activities of daily living. When discussing their lived experiences, our cohort echoed many common themes noted for other adults with IMD such as galactosemia and phenylketonuria [29–31]. Adults reported a shift in their priorities as they age. They have a better understanding of their MSUD and its management, but need more support related to adult-onset conditions, mental health, and long-term care planning. Health care transition was fragmented or unaddressed for all participants and many caregivers navigated this life stage without assistance from their metabolic team. Overall, our interviews demonstrated the variability of outcomes for adults with MSUD and the need to develop systems that provide adequate support for these individuals in adulthood.

One of the primary goals of this study was to describe current functional outcomes for adults with MSUD. Knowledge of the possibilities of adulthood with MSUD is helpful for affected individuals of all ages. Across all IMD, we lack information about long-term outcomes and are frequently only able to provide anecdotes about life expectancy, quality of life, and prognosis. This knowledge gap impedes our ability to properly counsel families. While we did not use MSUD history for purposive enrollment, all affected participants have classical MSUD and nearly all (16/19) were symptomatic at diagnosis. Thus, the long-term outcomes described do not appear to have a direct correlation to disease severity. This variability demonstrates our continued challenges in prognostication - some high-achieving adults had severe neonatal presentations or frequent crises in childhood. However, we are able to raise the ceiling on the possibilities of being an adult with MSUD. We should appreciate these possibilities and use them as motivation to develop systems that support their successes.

Our interviews demonstrate that adults are not just big children. Participants voiced changing priorities. There was less emphasis with diet adherence and greater concern about how the low protein diet recommended for MSUD will impact an aging body. Participants reported that it was nearly impossible to find clinicians who could integrate common medical complaints with rare disease knowledge, creating wariness around healthcare. For our participants, mental health had an increased influence on daily life with emotional stress becoming a significant trigger for metabolic decompensation. Participants described new symptoms of elevated leucine (brain fog, fatigue, headaches) that may not be elucidated by traditional metabolic questions. Yet, mental health emerged as a domain where transition planning was notably absent, despite caregivers and adults reporting that depression directly influenced dietary management and metabolic stability. Our participants were primarily older than 21 years - the age in the United States known as “the cliff,” where available services drastically disappear [5]. These repercussions were apparent in many interviews where affected adults lack support to balance their health with employment and relationships. While many are facing the demands of independence, neither adults nor their caregivers felt prepared for this life change. Unpreparedness stems partly from HCT protocols designed around dietary self-management, a domain where participants reported confidence, rather than the psychosocial and adult-onset medical complexities that increasingly dominate the lived experiences of adults with IMD.

Healthcare transition was limited and primarily remains diet-related [6, 22]. This emphasis may explain why dietary compliance decreased in importance for adults. It is possible that many years of routinely discussing dietary self-management instills greater confidence and skill. If true, this experience reinforces the role of the metabolism team in promoting healthcare transition. Subspecialty providers are increasingly recognized as the preferred arbiters of transition planning due to their specialized knowledge, and their strong therapeutic relationships [4, 32]. The multidisciplinary metabolism team is well formulated to provide holistic care to the young adult IMD population. Standardization of healthcare transition interventions will strengthen the metabolic provider’s role and ensure that topics other than diet receive similar dedication. Metabolic practices may benefit from formalizing their transition process using the steps outlined by the US’s Got Transition or the UK’s Ready, Steady, Go [8, 33]. However, IMD-specific adjustments will be needed. Metabolism practices may need both ambulatory- and hospital-based transition policies. The ambulatory policy could cover when patients are expected to speak with providers independently, outline transition milestones like scheduling appointments or calling for refills, and standardize discussions on educational, vocational, financial, and decision-making supports. A hospital-based policy would create standards around admissions including age limits for pediatric hospital admissions and a checklist for adult hospital admissions that recognizes the need for patients to bring their own formula and IMD-specific medications. These policies should be developmentally-appropriate and tailored to each individual’s abilities. Transition planning will vary internationally due to difference in clinician training models. In the US, metabolic clinicians are well positioned to promote transition readiness, because barring a revolution in the genetics workforce, they will likely be providing lifelong care to adults with IMDs. This longstanding care offers the opportunity to assess transition successes and revise policies to match patient needs. However, as transfer rarely occurs in the US, we should look to the UK and European experience on integrating adults with IMD into adult-centered healthcare [7, 34]. In these countries, adult metabolic clinics are staffed by physicians trained in adult medicine and metabolism [35]. Individuals with IMD must be prepared for an actual transfer from a pediatric to an adult-focused clinic. Examining the practices of dedicated adult metabolism clinics, including the facilitators and barriers to transfer, will provide necessary insight about setting goals and supports for adulthood.

At the risk of invoking all transition-related cliches, we must also address the notion of “little kids, little problems - big kids, big problems.” Our interviews show that many adults with MSUD are facing hard challenges that require conversations that can be awkward or uncomfortable. One of the most striking reports from this study is self-harm through dietary or medication noncompliance. Here, adults have successfully learned medical self-management, but leveraged this knowledge to deliberately provoke metabolic decompensation. Adults with MSUD are at high risk for depression and anxiety. Having a chronic illness also increases the risk of self-harm [36, 37]. These risk factors require screening at each ambulatory appointment. Metabolic providers should incorporate screening measures, such as the PHQ-9 for depression or the GAD-7 for anxiety. These scales should be enhanced by directly asking if mood has ever affected IMD management, due to the high risk of injury from noncompliance. Clinicians should be prepared to ask about other challenging topics including substance abuse, sexual activity, contraception, and safety. Predatory relationships, either sexual or financial, were a repeated concern among caregivers. Metabolic teams can be important advocates for our adult patients – by proactively encouraging adoption of decision-making support and financial protections and by regularly reviewing these supports. These topics may be less familiar to pediatrics-trained providers, the predominant pathway for metabolic physicians in the US. Thus, dedicated adult-centered metabolic training and a multidisciplinary approach with social work and case managers may be beneficial.

HCT does not occur in a vacuum and is a process for both patients and caregivers. Among pediatric-onset conditions, there is greater recognition that successful transition requires a partnership between patients, caregivers, and medical teams [38, 39]. While caregivers may take a step back during transition, they should be encouraged to be active supporters throughout the process and adulthood in general. The high levels of independence achieved by our participants were only possible through consistent caregiver support. Caregiver activities could be logistical - all adults who lived on campus during college routinely returned home to portion out a week’s worth of formula with parental assistance -- or it could be financial with parents subsidizing rent so an affected adult could live independently. While transition planning should encourage building skills for autonomy, it should also create an environment where a young adult feels comfortable asking for help. Teamwork is already an ethos of many metabolic practices and this should continue through adulthood.

This study has limitations. We were able to sample a small subset of adults with MSUD. Our purposive sampling aimed to include the range of intellectual and functional outcomes of MSUD. We did not include any affected individuals who were diagnosed through newborn screening, so our results may not be representative of the current adolescent MSUD population. Further, we did not obtain detailed medical histories from participants. Our previous study on adults with MSUD suggested that long-term outcomes cannot be directly inferred from clinical history alone [14, 20]. The goal of this study was to broadly describe aspects of being an adult with MSUD. While more people with MSUD are reaching adulthood, MSUD remains a rare disease and many clinicians are only familiar with their clinic population. Collecting the larger experience of adults with MSUD, even from a small number, helps universalize the condition. It also generates a starting point for defining transition- and adult-related priorities. Further, the themes identified in this study reflected previously reported themes for adults with phenylketonuria and galactosemia, including the need for greater independent living supports and poor transition planning. It is likely that many adult concerns are generalizable across different IMDs and coordinated efforts to improve long-term outcomes in these conditions should be disease-agnostic.

## Conclusions

While this study focused on adults with MSUD alone, we believe the results are generalizable for individuals with many other pediatric-onset IMDs and rare disease. People with IMDs are entering adulthood with many unanswerable questions about their long-term health and functional outcomes. They desire independence but lack clarity on the supports needed to obtain autonomy. Metabolic clinicians are well poised to lead transition planning, but need resources that standardize education and counseling. Ideally, these resources would be applicable across many IMDs and incorporate different developmental abilities. Having a better understanding of the long-term outcomes of these rare diseases is also crucial for identifying measurable outcomes for transition interventions. Newborn screening for treatable IMDs has been remarkably successful over the last 25 years. Standardized transition protocols informed by lived experiences and evolving priorities of adults with IMD, not predetermined clinical checklists, are needed to match the level of care that newborn screening has provided.

## Data Availability

De-identified data is available upon request to the corresponding author.

## Abbreviations

IMD: Inherited Metabolic Disorders
MSUD: Maple Syrup Urine Disease
AYA: Adolescents and young adults
FSG: Family Support Group
IEP: Individualized Education Plan

## References

[1] Sirrs S, Hollak C, Merkel M, et al. The Frequencies of Different Inborn Errors of Metabolism in Adult Metabolic Centres: Report from the SSIEM Adult Metabolic Physicians Group. JIMD Rep. 2016;27:85–91. 10.1007/8904_2015_435. PMC5580735 26450566

[2] Gariani K, Nascimento M, Superti-Furga A, Tran C. Clouds over IMD? Perspectives for inherited metabolic diseases in adults from a retrospective cohort study in two Swiss adult metabolic clinics. Orphanet J Rare Dis. 2020;15(1):210. 10.1186/s13023-020-01471-z. PMC7433045 32811506

[3] Lotstein DS, Kuo AA, Strickland B, Tait F. The transition to adult health care for youth with special health care needs: do racial and ethnic disparities exist? Pediatrics. 2010;126(Suppl):S129–36. 10.1542/peds.2010-1466F. 21123475

[4] Fair C, Cuttance J, Sharma N, et al. International and Interdisciplinary Identification of Health Care Transition Outcomes. JAMA Pediatr. 2016;170(3):205–11. 10.1001/jamapediatrics.2015.3168. PMC6345570 26619178

[5] Sandquist M, Davenport T, Monaco J, Lyon ME. The transition to adulthood for youth living with rare diseases. Children (Basel). 2022;9(5). 10.3390/children9050710. PMC9139297 35626888

[6] Gold Ji, Strong A, Gold NB, et al. The current state of adult metabolic medicine in the United States: results of a nationwide survey. Genet Med. 10.1016/j.gim.2022.04.018 PMC9911209 35543711

[7] Gold JI, Stepien KM. Healthcare transition in inherited metabolic disorders-is a collaborative approach between US and European centers possible? J Clin Med. 2022;11(19). 10.3390/jcm11195805. PMC9572070 36233672

[8] Got Transition. Transitioning to an adult approach to care without changing clinicians.

[9] Chace DH, Kalas TA, Naylor EW. The application of tandem mass spectrometry to neonatal screening for inherited disorders of intermediary metabolism. Annu Rev Genomics Hum Genet. 2002;3:17–45. 10.1146/annurev.genom.3.022502.103213. 12142359

[10] Ferreira CR. The burden of rare diseases. Am J Med Genet A. 2019;179(6):885–92. 10.1002/ajmg.a.61124. 30883013

[11] Langeveld M, Sirrs S, Schoenmakers DH, et al. Screening for Life: Perspectives From Adult Metabolic Specialists on Newborn Screening for Inherited Metabolic Diseases. J Inherit Metab Dis. 2025;48(4):e70057. 10.1002/jimd.70057. PMC12226250 40610367

[12] Kruszka P, Regier D. Inborn Errors of Metabolism: From Preconception to Adulthood. Am Fam Physician. 2019;99(1):25–32. 30600976

[13] Tchan M, Lehman A, van Dussen L, et al. The Frequencies of Different Inborn Errors of Metabolism in Adult Metabolic Centres: 10 Years Later, Another Report From the SSIEM Adult Metabolic Physicians Group. J Inherit Metab Dis. 2025;48(2):e70005. 10.1002/jimd.70005. 39912519

[14] Gold JI, Strong A, Gold NB, et al. Executive and adaptive function impacts long-term outcomes for adults with maple syrup urine disease. J Inherit Metab Dis. 2025;48(1):e12827. 10.1002/jimd.12827. 39655455

[15] Enns GM, Packman W. The adolescent with an inborn error of metabolism: medical issues and transition to adulthood. Adolesc Med. 2002;13(2):315–29. vii. 11986039

[16] Baruteau J, Brunetti-Pierri N, Gissen P. Liver-directed gene therapy for inherited metabolic diseases. J Inherit Metab Dis. 2024;47(1):9–21. 10.1002/jimd.12709. 38171926

[17] Musunuru K, Grandinette SA, Wang X, et al. Patient-Specific In Vivo Gene Editing to Treat a Rare Genetic Disease. N Engl J Med. 2025;392(22):2235–43. 10.1056/NEJMoa2504747. PMC12713542 40373211

[18] Gerstein MT, Markus AR, Gianattasio KZ, et al. Choosing between medical management and liver transplant in urea cycle disorders: A conceptual framework for parental treatment decision-making in rare disease. J Inherit Metab Dis. 2020;43(3):438–58. 10.1002/jimd.12209. PMC7318329 31883128

[19] Strauss KA, Carson VJ, Soltys K, et al. Branched-chain α-ketoacid dehydrogenase deficiency (maple syrup urine disease): Treatment, biomarkers, and outcomes. Mol Genet Metab. 2020;129(3):193–206. 10.1016/j.ymgme.2020.01.006. 31980395

[20] Muelly ER, Moore GJ, Bunce SC, et al. Biochemical correlates of neuropsychiatric illness in maple syrup urine disease. J Clin Invest. 2013;123(4):1809–20. 10.1172/JCI67217. PMC3613932 23478409

[21] Conditions Added to the Recommended Universal Screening Panel. Accessed September 12. 2025. https://www.hrsa.gov/advisory-committees/heritable-disorders/rusp/previous-nominations.

[22] Gold JI, Strong A, Gold NB, et al. Impact of executive and adaptive function on functional outcomes in adults with maple syrup urine disease. medRxiv. 2024;2024.05.17.24307509. 10.1101/2024.05.17.24307509.

[23] Packman W, Mehta I, Rafie S, Mehta J, Naldi M, Mooney KH. Young adults with MSUD and their transition to adulthood: psychosocial issues. J Genet Couns. 2012;21(5):692–703. 10.1007/s10897-012-9490-1. 22350623

[24] Robson C, McCartan K. Real World Research. 4th ed. Wiley; 2016.

[25] San Martin-Feeney D, Samborn S, Allemang B, et al. Transition experiences of adolescents and young adults working with a patient navigator. Heal care transitions. 2025;3:100088. 10.1016/j.hctj.2024.100088. PMC11658225 39712475

[26] Pierce JS, Aroian K, Schifano E, et al. Health Care Transition for Young Adults With Type 1 Diabetes: Stakeholder Engagement for Defining Optimal Outcomes. J Pediatr Psychol. 2017;42(9):970–82. 10.1093/jpepsy/jsx076. 28460055

[27] Elo S, Kyngäs H. The qualitative content analysis process. J Adv Nurs. 2008;62(1):107–15. 10.1111/j.1365-2648.2007.04569.x. 18352969

[28] Patton M. Qualitative Research and Evaluation Methods. Sage; 2002.

[29] Randall JA, Sutter C, Raither L, et al. Understanding the patient experience of Classic Galactosemia in pediatric and adult patients: increased disease burden, challenges with daily living, and how they evolve over time. J patient-reported outcomes. 2023;7(1):95. 10.1186/s41687-023-00635-2. PMC10522554 37751006

[30] O’Shea M-E, Sheehan Gilroy B, Greaney A-M, MacDonald A. Moving through adulthood: The lived experience of Irish adults with PKU. Front Psychol. 2022;13:983154. 10.3389/fpsyg.2022.983154. PMC9513515 36176784

[31] Frank N, Fitzgerald R, Legge M. Phenylketonuria–the lived experience. N Z Med J. 2007;120(1262):U2728. 17891216

[32] Varty M, Speller-Brown B, Phillips L, Kelly KP. Youths’ Experiences of Transition from Pediatric to Adult Care: An Updated Qualitative Metasynthesis. J Pediatr Nurs. 2020;55:201–10. 10.1016/j.pedn.2020.08.021. PMC7722194 32966960

[33] Graham-Rowe D. Ready, steady, go. New Scientist. 1999. Available from: https://www.nice.org.uk/sharedlearning/implementing-transition-care-locally-and-nationally-using-the-ready-steady-go-programme. Accessed 15 Apr 2022.

[34] Stepien KM, Kieć-Wilk B, Lampe C, et al. Challenges in Transition From Childhood to Adulthood Care in Rare Metabolic Diseases: Results From the First Multi-Center European Survey. Front Med. 2021;8:652358. 10.3389/fmed.2021.652358. PMC7962750 33738294

[35] Hannah-Shmouni F, Stratakis CA, Sechi A, et al. Subspecialty training in adult inherited metabolic diseases: a call to action for unmet needs. lancet Diabetes Endocrinol. 2019;7(2):82–4. 10.1016/S2213-8587(18)30369-3. 30683215

[36] Barnes AJ, Eisenberg ME, Resnick MD. Suicide and self-injury among children and youth with chronic health conditions. Pediatrics. 2010;125(5):889–95. 10.1542/peds.2009-1814. 20385631

[37] Ferro MA, Rhodes AE, Kimber M, et al. Suicidal Behaviour Among Adolescents and Young Adults with Self-Reported Chronic Illness. Can J Psychiatry. 2017;62(12):845–53. 10.1177/0706743717727242. PMC5714119 28814100

[38] Prussien KV, Crosby LE, Faust HL, et al. An Updated Equitable Model of Readiness for Transition to Adult Care: Content Validation in Young People With Sickle Cell Disease. JAMA Pediatr. 2024;178(3):274–82. 10.1001/jamapediatrics.2023.5914. PMC10775077 38190311

[39] Schwartz LA, Brumley LD, Tuchman LK, et al. Stakeholder validation of a model of readiness for transition to adult care. JAMA Pediatr. 2013;167(10):939–46. 10.1001/jamapediatrics.2013.2223. PMC4289606 23959392

